# Mathematical modelling of clostridial acetone-butanol-ethanol fermentation

**DOI:** 10.1007/s00253-017-8137-4

**Published:** 2017-02-16

**Authors:** Thomas Millat, Klaus Winzer

**Affiliations:** Clostridia Research Group, SBRC-Nottingham, a BBSRC/EPSRC Synthetic Biology Research Centre, School of Life Sciences, University of Nottingham, Nottingham, NG7 2RD UK

**Keywords:** Clostridial ABE fermentation, pH-induced metabolic shift, Mathematical modelling, Structural and dynamical models, Batch and continuous culture

## Abstract

Clostridial acetone-butanol-ethanol (ABE) fermentation features a remarkable shift in the cellular metabolic activity from acid formation, acidogenesis, to the production of industrial-relevant solvents, solventogensis. In recent decades, mathematical models have been employed to elucidate the complex interlinked regulation and conditions that determine these two distinct metabolic states and govern the transition between them. In this review, we discuss these models with a focus on the mechanisms controlling intra- and extracellular changes between acidogenesis and solventogenesis. In particular, we critically evaluate underlying model assumptions and predictions in the light of current experimental knowledge. Towards this end, we briefly introduce key ideas and assumptions applied in the discussed modelling approaches, but waive a comprehensive mathematical presentation. We distinguish between structural and dynamical models, which will be discussed in their chronological order to illustrate how new biological information facilitates the ‘evolution’ of mathematical models. Mathematical models and their analysis have significantly contributed to our knowledge of ABE fermentation and the underlying regulatory network which spans all levels of biological organization. However, the ties between the different levels of cellular regulation are not well understood. Furthermore, contradictory experimental and theoretical results challenge our current notion of ABE metabolic network structure. Thus, clostridial ABE fermentation still poses theoretical as well as experimental challenges which are best approached in close collaboration between modellers and experimentalists.

## Introduction

Acetate-butanol-ethanol (ABE) fermentation carried out by saccharolytic clostridia has attracted academic and industrial interest for over a hundred years. After the initial discovery of butanol formation by Louis Pasteur in 1862 (Pasteur 1862) and the subsequent discovery of acetone production by Chaim Weizmann in 1919 (Weizmann 1919), it quickly grew to commercial scale and became one of the largest industrial bioprocesses of the twentieth century. However, when petrochemical synthesis of solvents became economical in the 1950s and early 1960s (Jones and Woods 1986), industrial ABE plants in Western countries were closed down. The remaining facilities in South Africa and the Soviet Union terminated in the 1980s (Zverlov et al. 2006), and the last facility in China finally closed in 2004 (Chiao and Sun 2007). In the last decade, concerns over limited fossil resources and environmental impacts of petrol-based technologies have initiated the return of clostridial ABE fermentation to academic and industrial focus (Green 2011).

Natural ABE fermentation is exclusively performed by solventogenic clostridia. These strictly Gram-positive endospore-forming anaerobes possess a fermentative metabolism and utilize a wide variety of sugars, oligosaccharides and polysaccharides. Typically, their fermentation metabolism is characterized by two phases, exhibiting a distinct shift in the product spectrum. During acidogenesis, the main (liquid) products are acetic acid and butyric acid, whereas neutral solvents, i.e. butanol, acetone and ethanol, are the main fermentation products during the solventogenic phase. In both phases, hydrogen and carbon dioxide are formed as by-products.

Over the years, several solventogenic species have been isolated and characterized which differ in their substrate preferences, fermentation product profiles and other relevant properties. Common to all of them is the production of butanol and ethanol. In addition, *Clostridium acetobutylicum* (Jones and Woods 1986), *Clostridium saccharobutylicum* and *Clostridium saccharoperbutylacetonicum* (Keis et al. 2001) form acetone, whereas strains of *Clostridium beijerinckii* either produce acetone or reduce it further to isopropanol and *Clostridium aurantibutyricum* combines the formation of the two solvents (George et al. 1983). These two products are missing in *Clostridium tetanomorphum* (Gottwald et al. 1984). Similarly, *Clostridium puniceum* produces mainly butanol and comparatively little acetone and ethanol (Holt et al. 1988). Of the species mentioned here, only the first four have been used in industrial ABE fermentation (Shaheen et al. 2000).

Solventogenic clostridia also differ in genome size and organization. For instance, the ABE model organism *C. acetobutylicum* encodes several important solvent-forming enzymes on a large megaplasmid, pSOL1 (Nölling et al. 2001), whereas this information is located on the main chromosome in other solventogenic clostridia.

In this review, we summarize past and present attempts to model clostridial ABE fermentation. In contrast to other recent reviews on the subject (Mayank et al. 2013; Dash et al. 2016), we focus on the underlying biological, physicochemical and mathematical assumptions behind the proposed models, whether or not these are supported by experimental findings. Specifically, we restrict ourselves to ABE fermentation metabolism and the dynamic transition between acidogenic and solventogenic states although, undoubtedly, modelling-guided improvements in process design, including among others immobilized cells (Schoutens et al. 1986; Park et al. 1989; Badr et al. 2001; Vichuviwat et al. 2014), sophisticated bioreactors (Li et al. 2011b; Dolejš et al. 2014), product recovery (Xue et al. 2014) and life cycle assessments (Wu et al. 2008; Napoli et al. 2012), will all be crucial for a successful comeback of large-scale commercial applications. Furthermore, recent progress in genetic engineering of the ABE network will only be considered if the generated mutants have particular importance for a given model’s assumptions/predictions. We would like to emphasize that recent improvements in targeted mutagenesis of clostridia, e.g. using ClosTron (Cooksley et al. 2012; Kuehne and Minton 2012; Al-Hinai et al. 2012; Lütke-Eversloh 2014) or CRISPR (Wang et al. 2015; Li et al. 2016), offer new perspectives for modelling ABE fermentation.

We first introduce the relevant physiological basis of ABE fermentation. This is followed by an overview of the different modelling approaches employed. (We include only a minimal number of mathematical equations.) We then summarize and discuss published structural and dynamic models in their chronological order. Finally, we address open and emerging questions regarding experimental and theoretical investigations.

### Acetone-butanol-ethanol fermentation in solventogenic clostridia

Two different setups are widely used to investigate the reversible shift from acidogenesis to solventogenesis in both experiment and theory: batch culture and continuous culture.

**Fig. 1:**
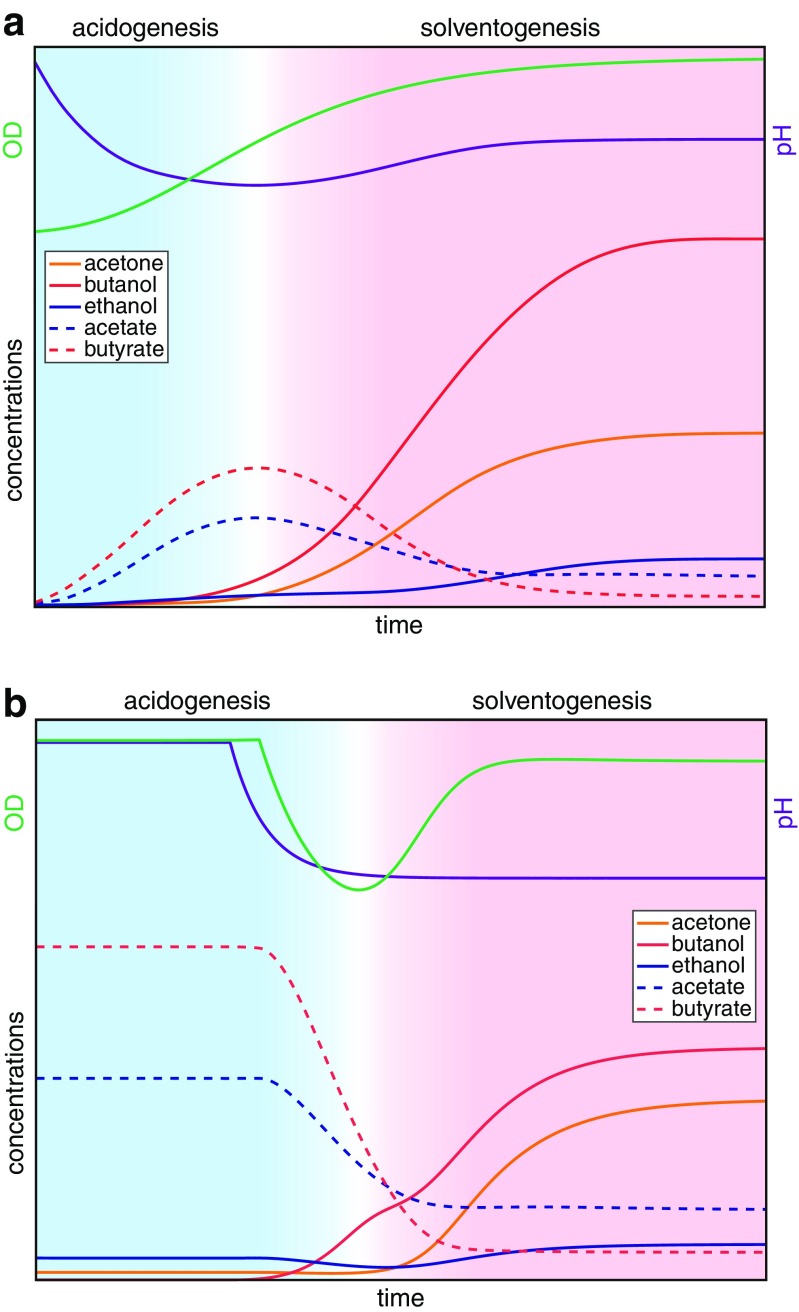
Traditional view of the changes in optical density, pH and product concentrations during a standard batch culture experiment (**a**) (Bahl et al.1982b) and forward shift experiment using a phosphate-limited continuous culture (**b**) (Millat et al. 2013a). Inoculation and initial growth phases not shown

During a standard batch fermentation as shown in Fig. 1a, the metabolic shift from acidogenesis to solventogenesis appears to be linked to the growth phase (Jones and Woods 1986). During early and mid-exponential phase, the cells produce acids to generate maximum ATP per substrate, which results in a pH decrease of the culture medium. As cell density increases further and the culture enters stationary phase, the metabolic shift to solvent formation is initiated. While sugar consumption continues, the cells start to re-assimilate the previously excreted acids resulting in an increase of the culture pH (Jones and Woods 1986). Additionally, the complex program of endospore formation is initiated (Alsaker and Papoutsakis 2005). From this observed temporal association of stationary phase, solvent formation and early stages of sporulation, one might conclude that solventogenesis is associated with non-growing cells and the onset of sporulation (see e.g. Schuster et al. 1998), but it is more likely that these processes share regulatory mechanisms (George et al. 1983; Ravagnani et al. 2000; Gottschal and Morris 1981; Harris et al. 2002) which are co-ordinately controlled by a master regulator such as Spo0A (Alsaker et al. 2004; Woolley and Morris 1990). Indeed, for some species and strains, solvent formation in batch culture has been reported to occur before their transition to stationary phase (Holt et al. 1988).

In continuous cultures under phosphate, nitrogen or iron limitation (Bahl et al. 1982a, b; Junelles et al. 1988), the reversible shift from acidogenesis to solventogenesis can be triggered by a change in external pH (Fig. 1b). For phosphate-limited chemostat cultures of *C. acetobutylicum* ATCC 824, the pH limits for this phase shift have recently been established (Millat et al. 2013b). A systematic variation of the external pH revealed that for pH >5.2, the cells favoured the formation of acids and, thus, established an acidogenic culture, whereas the production of solvents dominated for pH <5.1 which thus corresponded to the solventogenic phase.

Furthermore, experimental evidence suggests that nutritional composition (Robinson 1922; Bahl et al. 1986) and intracellular ATP and NAD(P)H pools (Girbal and Soucaille 1994; Wietzke and Bahl 2012) have a profound impact on product spectrum and temporal behaviour of ABE fermentation. Experiments indicate that the state of reduction of substrates and the redox potential of the medium are crucial to these findings (Kim et al. 1988) but also might be influenced externally using cell recycle (Hüsemann and Papoutsakis 1989) or by providing artificial electron donors (Kim and Kim 1988; Girbal et al. 1995).

Contradictory observations have been made for batch and chemostat setups (see Jang et al. 2014; Janssen et al. 2010). Similarly, continuous cultivation of *C. acetobutylicum* mutants defective in key fermentation genes has revealed significant phenotypic differences in comparison with the same mutants in batch culture (Hönicke et al. 2014), suggesting that the ABE fermentation network is more complex than previously thought and our understanding of the shift and the two metabolic states as well as the regulatory mechanisms used to achieve them remains incomplete.

Solventogenic clostridia are unable to maintain a constant intracellular pH, and as a result, changes in the external pH have a direct impact on the intracellular biochemical and biophysical conditions. Under normal physiological conditions, the transmembrane pH gradient is kept constant with an approximate difference of one pH unit (Gottwald and Gottschalk 1985; Huang et al. 1985). Hence, mathematical models of clostridial ABE fermentation should consider pH-dependent factors as important variables.

**Fig. 2:**
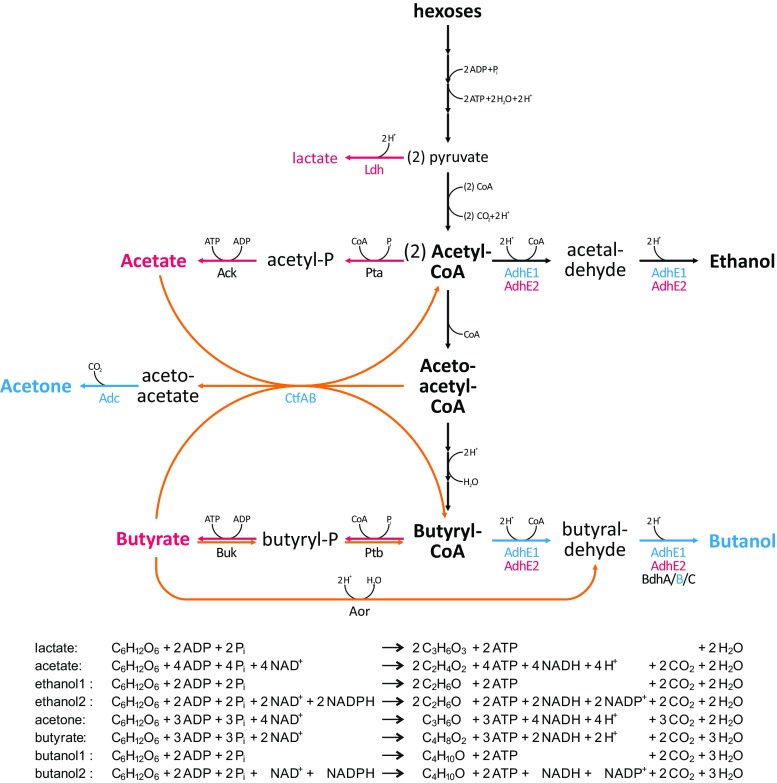
The metabolic network of ABE fermentation in wild-type *C. acetobutylicum* (Jones and Woods 1986). During acidogenesis (high pH), the culture predominantly forms the organic acids acetate and butyrate (*red*), whereas solventogenesis (low pH) features the formation of the solvents acetone and butanol (*blue*). Ethanol is formed in minor but similar amounts during both metabolic phases. In particular, solvent-forming enzymes are subject to state-dependent synthesis as indicated by the *different colours*. Three different mechanisms for acid re-assimilation (*orange*) are considered in the models discussed in this review: (1) acetate and butyrate cycles consisting of CtfAB-dependent acid assimilation and ATP-forming acid kinase reactions coupled to acetone formation (Hartmanis et al. 1984); (2) a reverse Buk-Ptb mechanism (Desai et al. 1999a); and (3) Aor-dependent re-utilization (Millat et al. 2014). The chemical equations below the network summarize the mass balances resulting from stoichiometric conversion of glucose to the indicated products. Two equations are given for the formation of alcohols since aldehyde/alcohol dehydrogenases (AdhE) use NADH as cofactor, whereas butanol dehydrogenases (Bdh) use NADPH. Note that the function of AdhE1 as alcohol dehydrogenase has been challenged (Yoo et al. 2015) (colour figure online)

The principal metabolic network of the ABE fermentation (see Fig. 2), is well understood (Jones and Woods 1986). An extensive summary of the involved enzymes, their biochemical properties and proposed reaction mechanism can be found in Gheshlaghi et al. (2009). Solventogenic clostridia possess several phosphoenolpyruvate-dependent phosphotransferase systems (PTSs) for the uptake of various carbohydrates (Nölling et al. 2001; Mitchell and Tangney 2005). However, glucose is a preferred substrate and activates catabolic repression systems to prevent simultaneous uptake and utilization of other sugars (Patakova et al. 2013). Interestingly, solventogenic cells exhibit a lower PTS activity compared to acidogenic cells (Hutkins and Kashket 1986), but no significant differences in glucose consumption have been observed experimentally (Jang et al. 2014). Glycolytic breakdown of internalized hexoses conserves energy in the form of two ATPs, yielding two molecules of acetyl-CoA, two CO_2_, two NADH and one reduced ferredoxin (per molecule hexose converted). The latter can serve as a substrate for hydrogenase, thus liberating hydrogen.

Three key branch points in the ABE network direct the metabolic flux towards either acids or solvents. First, acetyl-CoA serves as the starting point for the formation of acetate (+1 ATP), ethanol and acetoacetyl-CoA (via condensation). Second, acetoacetyl-CoA is a precursor for acetone and butyryl-CoA. Third, butyryl-CoA can be converted to butyrate (+1 ATP) and butanol. As mentioned above, after the initiation of solventogenesis, the formerly excreted acids are re-assimilated and converted to their respective CoA derivatives in reactions that involve CtfAB CoA transferase (Andersch et al. 1983; Hartmanis et al. 1984) and possibly other mechanisms (Desai et al. 1999a; Millat et al. 2014) as depicted in Fig. 2.

Under certain conditions, lactate can also be formed, for instance when hydrogenase activity is inhibited by carbon monoxide (Datta and Zeikus 1985; Kim et al. 1984) or due to iron limitation (Bahl et al. 1986; Peguin and Socaille 1995). However, the lactic acid pathway is not operational under normal conditions and generally inactive under solventogenic pH levels (Jones and Woods 1986).

From this short summary and the fermentation scheme shown in Fig. 2, it is clear that solventogenic cells conserve less ATP per molecule glucose than acidogenic cells. It should be noted that *C. acetobutylicum*, in contrast to other solventogenic clostridia, does not possess an RNF complex and may therefore be unable to harness the energy released in the electron transfer from reduced ferredoxin to NAD (Biegel et al. 2011; Buckel and Thauer 2013). Thus, ATP generation via substrate-level phosphorylation appears to be the only mechanism of energy conservation in this organism.

Recent genome-wide studies have revealed that the metabolic shift from acidogenesis to solventogenesis is accompanied by considerable transcriptomic changes, e.g. Wang et al. (2012) and Wang et al. (2013) (batch culture) and Grimmler et al. (2011) and Janssen et al. (2012) (continuous culture), and proteomic cellular composition, e.g. Jang et al. (2014) (batch culture) and Janssen et al. (2010) (continuous culture). Generally, production of solvent-forming enzymes is induced and that of acid-forming enzymes reduced, although several exceptions have been reported (Jang et al. 2014; Janssen et al. 2010). Furthermore, several genes display complex expression patterns during the transition from acid to solvent formation (e.g. Grimmler et al. 2011), suggesting that they are subject to complex genetic regulation.

Importantly, several key enzymes possess significant pH-dependent activities under physiological conditions, thus imposing kinetic regulation on ABE fermentation. As may be expected, key solvent-forming enzymes generally display an increased kinetic activity under solventogenic conditions, whereas that of acid-forming enzymes is reduced under these conditions (Andersch et al. 1983).

Without doubt, the transition from acidogenesis to solventogenesis requires drastic changes at all levels of cellular organization (and potentially even the population level). Insight into the biological principles causing these changes can be gained through the generation of testable hypotheses using mathematical models. A discussion of various approaches towards the mathematical description of clostridial ABE fermentation is given in the following sections.

### Models

Existing models of ABE fermentation can be classified according to different schemes. In this review, we group them according to the employed theoretical framework and, where applicable, the experimental setup, i.e. batch or continuous culture. This approach allows us to trace the development of different models from their first implementation, including the latest developments. However, we do not aim to provide a detailed description of each model and its theoretical basis; instead, we only sketch major features and refer to the respective publications for more information.

Importantly, most models available to date share a common starting point assuming that changes in population-wide metabolite concentrations are determined by the network structure, the speed of the biochemical conversions and transport processes and the population size, but differ in how they proceed further. This kinetic approach is commonly expressed in matrix form (Heinrich and Schuster 1996):

1 $$ \left[\begin{array}{c}\hfill \frac{dx_1}{dt}\hfill \\ {}\hfill \vdots \hfill \\ {}\hfill \frac{dx_m}{dt}\hfill \end{array}\right]=\mathcal{N}\cdot \left[\begin{array}{ccc}\hfill {s}_{11}\hfill & \hfill \cdots \hfill & \hfill {s}_{1 n}\hfill \\ {}\hfill \vdots \hfill & \hfill \ddots \hfill & \hfill \vdots \hfill \\ {}\hfill {s}_{m1}\hfill & \hfill \cdots \hfill & \hfill {s}_{m n}\hfill \end{array}\right]\cdot \left[\begin{array}{c}\hfill {\nu}_1\hfill \\ {}\hfill \vdots \hfill \\ {}\hfill {\nu}_n\hfill \end{array}\right]- D\cdot \left[\begin{array}{c}\hfill {x}_1\hfill \\ {}\hfill \vdots \hfill \\ {}\hfill {x}_m\hfill \end{array}\right]. $$

Here, the matrix on the left hand side describes the population-wide changes in metabolite concentration per time. The first matrix on the right hand side is the stoichiometric matrix ***S***, which represents the metabolic network of *n* reactions and *m* metabolites. Its elements are negative or positive, representing the number of molecules consumed or produced, respectively, in a given reaction. An appropriate determination of this matrix is crucial for the later solution of the matrix equation and strongly depends on available biological information and experimental data but also the research questions under consideration.

The stoichiometric matrix ***S*** is multiplied by the flux vector ***ν*** that encodes for reaction rates or fluxes. Its elements describe the frequency of a metabolic reaction that depends on several influencing factors including enzyme activity and abundance of enzyme, substrate(s) and inhibitors. All these factors are subject to changes by cellular regulation and experimental setup. Furthermore, the frequency is restricted by physicochemical and biological constraints, which can be used to define the space of solutions but also to validate the found solution.

In addition, we explicitly include the population size  into the above matrix equation to emphasize that the population-wide metabolite concentrations are a function of the cellular activity and the number of cells in the population. The population size can be taken into account using either any observable quantity that is proportional to the number of cells, such as dry mass and optical density, or a growth law, e.g. exponential growth.

The second term in Eq. (1) considers the dilution of metabolites *x*_*i*_ in continuous culture. For the sake of simplicity, we have assumed that the dilution rate *D* is constant in time and for every metabolite. However, if retention or product-specific extraction systems, e.g. membranes, are applied, the dilution rate transforms into a matrix with metabolite-specific elements.

Major differences exist between the various models as to how the matrix equation is solved for a given biological system or problem. In very general terms, existing approaches focus either on the left or right hand side of Eq. (1) by simplifying the respective opposite side.

Dynamic models intend to explain temporal changes, i.e. the matrix on the left hand side, based on assumptions about reaction rates, including reaction mechanism and cellular regulation. Consequently, these models are well suited to test hypotheses with respect to putative reactions and their mechanisms as well as their regulation. However, their high demand on dynamic data and additional information restrict the feasible network size, i.e. matrix ***S***.

In contrast, structural models use information about the network structure to investigate the metabolic capabilities of the cells. Towards this end, a (quasi-)steady assumption is applied that transforms the system of differential equations into a system of algebraic equations. Then, flux balance analysis (FBA) (Kauffman et al. 2003; Orth et al. 2010; Varma and Palsson 1994) and elementary mode analysis (EMA) (Schuster et al. 1999; Schuster and Hilgetag 1994; Heinrich and Schuster 1998) are applied to determine a matrix ***ν*** that fulfils the resulting equation

2 $$ 0=\boldsymbol{S}\cdot \boldsymbol{\nu} $$

and provides insights into the metabolic routes and their activities during the metabolic (steady) states. Accordingly, these models are used to investigate alterations to the bacterial metabolism in response to changes in the metabolic network.

### Structural models for solventogenic clostridia using flux balance analysis

Flux balance analysis (FBA) assumes that the metabolism approaches a steady state that is determined by the metabolic network (stoichiometry) including enzymatic reactions, transport processes, physicochemical properties and biological constraints such as toxicity and ATP maintenance costs (Kauffman et al. 2003). Generally, our incomplete knowledge of these constraints, in particular the physicochemical ones, leads to underdetermined systems for which multiple steady-state solutions exist. Hence, an optimization is carried out to find an optimal distribution of fluxes with respect to a specified (multivariate) objective function (Segrè et al. 2002). However, it should be noted that regulation of reactions or pathways is not explicitly considered.

The first model employing the idea that metabolic activity of solventogenic clostridia at steady state balances elemental composition of utilized organic substrates, microbial biomass and extracellular products was published by Papoutsakis in 1984 (Papoutsakis 1984). It uses stoichiometric equations to represent the conversion of glucose into primary products and key intermediates of ABE fermentation and, thus, is often referred as ‘stoichiometric’ model. From the system of stoichiometric equations, a fermentation equation is derived that describes the flux distribution towards the formation of included metabolites, energy carriers and biomass. The respective fluxes and, thus, the coefficients of this equation are constrained by the necessary balance of carbon, nitrogen, hydrogen and oxidation/reduction reactions and by assuming irreversible metabolic reactions, except for the formation of NADH and reduction of ferredoxin.

Based on the derived fermentation equation, Papoutsakis (1984) found that while acetone, isopropanol, butyrate and acetate could each be formed as the sole product in an ideal fermentation, the formation of butanol required simultaneous formation of acetate. The computed product yields represented theoretical maxima of product formation to achieve in ABE fermentation from a given sugar, since the ‘simple’ stoichiometric network neglected other cellular functions. This approach can be considered a valid starting point for the evaluation of the potential of a given metabolic network for biotechnological applications.

In this first model, the re-assimilation of acetate during the metabolic shift and solventogenesis was neglected. However, while this approach agreed with experimental studies suggesting that it is mainly butyrate that is re-utilized under both batch and continuous culture conditions (Mermelstein et al. 1993; Millat et al. 2013a), it was at odds with biochemical evidence showing that clostridial CtfAB CoA transferases prefer acetate to butyrate as a substrate (Wiesenborn et al. 1989). Furthermore, addition of exogenous acetate to the culture medium was reported to have a positive effect on solvent formation in solventogenic clostridia (Gao et al. 2016; Fond et al. 1985; Chen and Blaschek 1999a) suggesting an active pathway for reutilization of acetic acid.

Hence, Desai and Papoutsakis in 1999 (Desai et al. 1999b) extended the original stoichiometric model by adding a CoA transferase-dependent pathway for acetate re-utilization. However, this addition resulted in a singularity, i.e. no unique solution could be found for the network during solventogenesis. This singularity is caused by the simultaneously active pathways of formation and re-assimilation of acetate and butyrate, respectively (Fig. 2). Since both ‘acid cycles’ independently generate ATP by cycling the acids, maximal ATP generation could be achieved by multiple combinations of differently active acetate or butyrate cycles.

To resolve this singularity, the authors introduced an additional, non-linear constraint that relates the experimentally observed ratio of butyrate and acetate uptake to the ratio of their intracellular concentrations. The latter were assumed to be constant and identical to the ratio of external concentrations. In this form, the model allowed determination of a unique metabolic flux distribution that reproduced the experimentally observed rates of acid uptake and product accumulation. It should be noted, however, that the internal and external butyrate/acetate ratios are approximately constant (though not identical) for solventogenic pH values only.

Finally, the authors concluded from the calculated production and reutilization rates of acetate, butyrate and acetone that acetate re-assimilation primarily accounted for acetone production, while butyrate was re-assimilated through both the reverse butyrate and acetone pathways (see Fig. 2). Furthermore, they predicted that a considerable amount of acetate is continuously formed and to a large degree cycled to generate additional ATP during solventogenesis. These findings suggested that acid formation plays a significant role for ATP generation in both metabolic phases.

In a follow-up study (Desai et al. 1999a), the authors applied their extended model to batch fermentation data obtained for a strain overproducing the enzymes required for butyrate formation, phosphotransbutyrylase (PTB) and butyrate kinase (BUK), and a mutant deficient in the *buk* gene. Interestingly, analysis of the calculated metabolic fluxes suggested that butyrate re-utilization is decoupled from acetone formation, again implying the existence of a CtfAB-independent route for its re-assimilation.

Whereas the metabolic networks discussed above were manually constructed based on the biochemical and genetic information available at the time, the early 2000s experienced the rise of database-driven network reconstruction. This development was initiated by major improvements in sequencing technologies allowing for an ever increasing number of organisms to be completely sequenced. As a result, the annotated genomes for *C. acetobutylicum* ATCC 824 and *C. beijerinckii* NCIMB 8052 were published in 2001 and 2011, respectively (Nölling et al. 2001; Wang et al. 2011), and have since served as a basis for reconstruction of metabolic reactions and membrane transport processes related to ABE fermentation. The genomes of *C. saccharobutylicum* and *C. saccharoperbutylacetonicum* have also become available (Poehlein et al. 2013; Poehlein et al. 2014), but have not yet been exploited for modelling purposes. Additionally, recent re-sequencing of *C. acetobutylicum* ATCC 824 (Ehsaan et al. 2016) found several discrepancies from the aforementioned genome sequence (Nölling et al. 2001) with not yet known consequences for the reconstruction of its metabolic network.

To start with, genome-scale (metabolic) models use information from annotated genome sequences to reconstruct a first draft metabolic network. Then, missing and incorrect reactions are identified resulting from incomplete, inaccurate and ambiguous annotation. Thermodynamic information is used to categorize the metabolic reactions with respect to their direction and to determine the overall feasibility of the network. Furthermore, cellular information, e.g. biomass composition, cellular maintenance costs and biochemical information such as metabolite fluxes, constrain possible steady-state solutions (see reviews (Fell et al. 2010; Thiele and Palsson 2010; Durot et al. 2009) for more details). Because of their fundamental design principle, genome-scale models do not include internal regulation, but their results could be considered by imposing external constraints based on experimental observations. Many automated tools have been developed to support the many construction steps, but still significant manual validation is required on the way towards reliable genome-scale networks.

The first two genome-scale metabolic models of *C. acetobutylicum* were established simultaneously in 2008 by Senger and Papoutsakis (2008a, b) and Lee et al. (2008). Interestingly, different solutions were proposed to fill gaps in the reconstructed metabolic network caused by incomplete functional information about genes and their products. More specifically, both attempts used mutually exclusive mechanisms for the *α*-ketoglutarate biosynthesis. In Senger and Papoutsakis (2008a), it was suggested that the urea cycle is used to compensate for an incomplete TCA cycle, whereas Lee et al. (2008) assumed that the reductive pathway from pyruvate to *α*-ketoglutarate is connected. However, recent ^13^C-labelling experiments corroborated the first assumption (Au et al. 2014).

In their first paper, Senger and Papoutsakis (2008a) also assessed the thermodynamic feasibility of proposed pathways under acidogenic and solventogenic conditions. Towards this end, the authors assumed that a negative Gibbs free energy of reaction is required for every metabolic reaction in a pathway under physiological conditions (except the assumption of neutral pH) to render a pathway thermodynamically feasible. Most interestingly, their analysis using l-glutamate as an example suggested that changing ADP/ATP and NAD^+^/NADH ratios (Girbal and Soucaille 1994; Grupe and Gottschalk 1992; Meyer and Papoutsakis 1989), which in turn depend on the metabolic state of the culture, could alter the thermodynamic feasibility of pathways and, thus, could initiate an adaptation in the cellular metabolic program.

In a subsequent paper (Senger and Papoutsakis 2008b), the authors focussed on changes to the specific proton flux during acidogenesis and solventogenesis. Towards this end, extracellular proton concentrations and their transport across the cell membrane were introduced into the model. According to this extended model, acid- and solvent-forming cells differ considerably in terms of their proton fluxes, suggesting that this trait may be used to identify the prevailing metabolic program and, hence, phenotypic composition of a culture. Interestingly, for the extended model to accurately reproduce the experimentally determined extracellular pH changes in batch culture, multiple discrete proton flux states had to be assumed, each of which might represent a distinct cellular state. According to this interpretation, acid and solvent batch culture populations would consist of more than two distinct metabolic cell types.

By contrast, Lee et al. (2008) used their model to investigate the impact of maintenance energy on growing (acidogenic) and non-growing (solventogenic) cells. Consistent with experimental data, it was shown for growing cells that an increased demand for maintenance energy led to a decrease in specific growth rate and simultaneously shifted acid formation from butyrate to acetate. Furthermore, the model predicted an elevated butanol production under inhibited hydrogenase activity. An additional model analysis identified essential genes providing information about genetic targets for metabolic engineering (Brockman and Prather 2015).

Starting from the model by Senger and Papoutsakis (2008a, b), McAnulty et al. (2012) constructed a new, considerably extended model (*i*CAC490). Contrary to previous models, it contains a full TCA cycle considering recent fluxomics data (Crown et al. 2011; Amador-Noguez et al. 2010). Furthermore, a new method (FBrAtio) that considers how a metabolite pool (branched metabolite) is distributed among competing enzymes and, thus, fluxes introduced additional constraints to the metabolic network. Previously estimated ratios (Desai et al. 1999b) were applied to describe the *C. acetobutylicum* wild type. Moreover, several combinations of CtfAB CoA transferase and (supposedly) bifunctional AdhE knockdowns and overexpressions were analysed with respect to changes in product formation.

More recently, Dash et al. (2014) reported the construction of a second-generation genome-scale model of *C. acetobutylicum* (referred to as *i*Cac802). Taking into account new results from labelling experiments (Au et al. 2014), this model again considered an incomplete (branched) TCA cycle. However, the reported experiments (Au et al. 2014) used a defined media supporting significantly slower growth than complex media used in fluxomics experiments (Crown et al. 2011; Amador-Noguez et al. 2010) which might result in different flux distributions. Furthermore, the model included a complete fatty acid synthesis pathway and additions to the purine, pyrimidine and cobalamin biosynthetic pathways. Moreover, several thermodynamically unfavourable reactions were re-inserted and related reactions were either removed or altered to avoid the formation of thermodynamically infeasible cycles based on ^13^C-labelling experiments (Au et al. 2014). The resulting network included around 800 genes, 1500 reactions and 1200 metabolites and was thus three times larger than the first-generation models (Senger and Papoutsakis 2008a, b; Lee et al. 2008). Finally, the model was calibrated using calculated fluxes recently published by Lehmann et al. (2012).

Additionally, the authors (Dash et al. 2014) established a method referred to as CoreReg to incorporate regulatory information into structural models. CoreReg modified the flux bounds of reactions according to fold changes in gene expression which were experimentally observed under butyrate and butanol stress conditions (Wang et al. 2013). Subsequent interrogation of the model corroborated previous experimental findings (Wang et al. 2013; Alsaker et al. 2010), i.e. butanol stress strongly influences arginine metabolism, whereas butyrate stress imposes regulations on reactions in arginine and pyrimidine metabolism.

In a very recent study, Yoo et al. (2015) combined a further improved genome-scale model with quantitative transcriptomic, proteomic and fluxomic data. With a total of 967 genes, this model, referred to as *i*Cac967, considered 20% more genes than *i*Cac802 (Dash et al. 2014), but incorporated only 84% of the latter’s reactions and 93% of its metabolites, illustrating that proposed gene functions are far from common consensus and the need for manual, expert curation of automated annotations.

The study included biochemical characterizations of several key solvent-forming enzymes. While the bifunctional role of AdhE2 as a butanol/butyraldehyde dehydrogenase was confirmed (Fig. 2), AdhE1 was found to lack significant butanol dehydrogenase activity, thus requiring the presence of additional butanol dehydrogenase(s) to form butanol from butyryl-CoA. Furthermore, the authors reported that the activity of all three butanol dehydrogenases (BdhA-C) depends on NADPH and not NADH as claimed in previous biochemical studies (Welch et al. 1989; Petersen et al. 1991).

Another key aspect of this study was the combination of the developed genome-scale model with quantitative transcriptomic and proteomic data obtained from phosphate-limited continuous cultures (Yoo et al. 2015) to determine metabolic fluxes under acidogenic and solventogenic conditions. Interestingly, this approach confirmed suggested distinct roles for (alcohol)/aldehyde dehydrogenase AdhE1/2 (Grimmler et al. 2011; Millat et al. 2013a), with AhdE2 being crucial for butanol formation under acidogenic conditions and AdhE1 together with the major butanol dehydrogenase BdhB under solventogenic conditions.

The first genome-scale model, *i*CM925, for *C. beijerinckii* NCIMB 8052 was published by Milne and co-authors in 2011 (Milne et al. 2011). This model considered 925 genes, 938 reactions, 881 metabolites and 67 membrane transporters. Simultaneously to the construction of the model, the authors conducted batch culture experiments at different temperatures and determined growth rate, substrate uptake (glucose and acetate) and secretion of liquid products. The latter experimental information was applied to further constrain the model. From the deviations found between their model predictions using optimal growth as sole criterion and their experimental observations, the authors finally concluded that additional selection pressures gave rise to the observed behaviour. Interestingly, no metabolic flow towards and from malate was found in the model rendering the structural complete TCA cycle functionally incomplete under the considered experimental conditions. Furthermore, the authors investigated the effect of varying fixed hydrogen production on the model behaviour. It was found that high hydrogen production forces the metabolic flow towards acetate whereas low levels of hydrogen production favours butanol formation which is in line with different amounts of hydrogen experimentally observed during acidogenesis and solventogensis (Rogers 1986).

Starting with small stoichiometric models and eventually using more and more detailed metabolic representations on the genome scale, flux balance analysis and subsequent non-linear optimization have been established as an integral method for the theoretical investigation of ABE fermentation. The different modelling attempts discussed above provided valuable knowledge about how the metabolic fluxes alter at acidogenic and solventogenic conditions and how mutations divert those fluxes towards differing product spectra. Perhaps somewhat surprisingly, key parts of the metabolic network are still a matter of debate, in particular whether a TCA cycle is operational or not, and how changing environmental conditions alter the network structure, e.g. by alteration of thermodynamic feasibility of reactions. Importantly, genome-scale models do not consider existing regulatory mechanisms and therefore cannot describe metabolic transitions as a cascading sequence of events.

### Structural models for solventogenic clostridia using elementary mode analysis

In elementary mode analysis, the steady-state capabilities of the metabolic network are represented as a set of irreducible pathways such that their linear combination (superposition) results in the experimentally found metabolic fluxes (Schuster et al. 1999; Schuster and Hilgetag 1994; Heinrich and Schuster 1998). Here, an irreducible pathway consists of a minimal set of biochemical reactions and transport processes at steady state that cannot be decomposed into simpler (sub)networks without loss of functionality. A combinatorial explosion in the number of elementary modes with increasing network size renders their computation an intricate numerical problem, but over the last decade, several approaches have been developed to tackle this challenge (see e.g. Schuster et al. 2000a *;* Wagner 2004; Klamt et al. 2005). Finally, the identified elementary modes are weighted by the flux they carry such that their linear combination matches experimentally determined rates of substrate uptake and product formation (Heinrich and Schuster 1996). Again, the solution space is restricted by physicochemical and biological constraints and optimality criteria select for specific solutions.

In 2014, Kumar et al. (2014) applied this approach to model the metabolic fluxes in *C. acetobutylicum* under batch culture conditions for acidogenesis, solventogensis and stressed conditions. Interestingly, the authors found three different classes of modes: first, acidogenic modes that exhibit glucose consumption, acid formation and biomass production; second, solventogenic modes that form solvents using either glucose or glucose together with re-assimilated acids; and third, mixed modes that form acids and solvents concomitantly, but differ in biomass production. Importantly, no modes were found that resulted in glucose-independent solvent formation. However, some of the reported findings disagree with previous experimental work using mutants of *C. acetobutylicum*. For instance, the authors found for acidogenic modes that generation of butyrate was essential for biomass formation, even though butyrate-negative *ptb* mutants had already been generated and reported to display elevated acetate and ethanol production (Cooksley et al. 2012; Lehmann et al. 2012). Furthermore, the authors found that butanol and acetone formation is strictly coupled, but recent experiments using a *ctfA* mutant revealed that significant butanol production can take place even in the absence of acetone formation (Millat et al. 2014).

Furthermore, the authors used experimental data for stressed and unstressed cells (Kumar et al. 2013) to study changes in activity of elementary modes due to altered external conditions. Interestingly, the authors showed that solventogenesis in *C. acetobutylicum* is not causally linked to stationary growth in batch culture as often claimed in the literature (e.g. Alsaker and Papoutsakis 2005; Desai et al. 1999a; Grimmler et al. 2011; Lee et al. 2008; McAnulty et al. 2012). As might be expected, the pattern of found elementary mode activity revealed that acidogenic modes dominate during acidogenesis and solventogenic modes during solventogenesis. Under stressed conditions (e.g. a sudden change of external pH and addition of exogenous acids), elementary modes became active that combined acidogenic and solventogenic characteristics resulting in a culture that exhibited the two featured fermentations states simultaneously. In agreement with previous proposals (Clarke et al. 1988), it was suggested that this could be explained by the occurrence of heterogeneous subpopulations.

A decomposition into elementary modes provides a set of metabolic pathways that can be classified according to a given feature, e.g. products, commonly used enzymes or metabolic activity at given conditions, an information which can be used to deliberately target specific groups of pathways by means of metabolic engineering. Unfortunately, the number of elementary modes is rapidly increasing with the network size rendering a targeted analysis difficult or even intractable. However, current research is ongoing to reduce the number of elementary modes based on biological and physicochemical criteria (Hartman et al. 2014; Gerstl et al. 2016).

### Structural models for solventogenic clostridia using graph theory

An alternative approach to the stoichiometric matrix used for flux balance analyses and elementary mode analysis discussed above, signal flow graphs were recently employed by Li et al. (2013, 2014) to model the carbon flow through a simplified ABE network without ethanol formation. Considering metabolic networks, a signal flow graph is a directed graph which represents a unidirectional molecular flow. Its vertices (or nodes) represent signals, e.g. metabolite concentrations, and the edges (branches, arrows) represent functional dependencies, e.g. carbon flow from metabolite to metabolite (Mason 1956). A vertex receives signals via incoming edges and transmits information along outgoing edges, information being, for instance, increases or decreases in the concentration of a given metabolite. Thus, the net signal of each node is the sum over all incoming and outgoing edges. Taken together, these components establish a set of algebraic equations describing the vertices of the network (Mason 1956; Dobrijevic et al. 1995).

This set of equations is represented in matrix form similar to Eq. (1), where the matrix on the left hand side now represents the signals, i.e. metabolite concentrations. Two related matrices can be used to represent the network structure (Diestel 2005). The adjacency (or connection) matrix describes whether two vertices are connected or not, whereas the incidence matrix specifies whether a vertex depends upon an edge. The latter matrix is generally used to represent physical systems, because it evidently maps cause and effect. For directed graphs, its elements are ±1 representing an incoming or an outgoing edge, respectively, and zero for unrelated combinations. Thus, an incidence matrix is similar to a stoichiometric matrix, but not equivalent as it excludes stoichiometric factors. Instead, stoichiometric factors are included in signal transfer functions that define the signal flow along an edge. Analogous to Eq. (1), the matrix of signal transfer functions is multiplied with the incidence matrix. Importantly, rules can be applied to construct signal transfer functions for network paths (Mason 1956).

Applying this theoretical framework, Li et al. (2013) investigated the factors which determine the acetone/butanol ratio and the formation of NADH. The authors found that the acetone/butanol ratio was primarily affected by the butyrate cycle (Fig. 2) with an attenuated activity of this cycle predicted to improve butanol formation. This finding is consistent with the experimentally observed reduced specific activity of butyrate kinase during solventogenesis (Andersch et al. 1983).

In a follow-up study (Li et al. 2014), signal flow graphs were also employed to model the effects of feeding exogenous acetate or butyrate during solventogenesis which, in accordance with previous experimental evidence (Chen and Blaschek 1999a, b), revealed a positive influence of either acid on solvent formation. However, as the original model (Li et al. 2013) could not fit experimental data sufficiently, its structure was revised by taking into account the altered experimental conditions. It was found that a modified model with incomplete acid cycles agreed best with data obtained in acid feeding experiments (Li et al. 2014). Hence, the authors speculated that acid cycle disruption (Fig. 2) is caused by unfavourable thermodynamic conditions resulting from acid addition.

The presented results suggest that signal flow graphs might be an interesting alternative to the kinetic approach Eq. (1) and appears to be particularly promising in situations where the kinetic mechanisms are unknown. However, the studies discussed above did not consider cellular alterations between the two metabolic phases. It would be interesting to identify changes to the network, signal transfer functions and/or network parameters, in particular distribution coefficients of branch points, which occur during the transition. Such an investigation could provide additional information to both structural and dynamical models.

### Dynamic models

In contrast to the structural models discussed above, dynamic models of ABE fermentation describe the changes of key metabolites in clostridial metabolism over time. Naturally, the dynamic transition between acidogenesis and solventogenesis and the intra- and extracellular events triggering this response are the focus of these approaches. Additionally, sensitivity analyses investigating the effect of kinetic parameters and gene expression on product formation often complement these studies. Towards this end, dynamic models consider biochemical and biophysical mechanisms as well as cellular regulations relevant to the ABE network. The activity (flux) of the network is determined by the abundance of participating or affecting molecules (e.g. substrates, enzymes, cofactors, products and inhibitors) and kinetic parameters describing molecular details of the processes (e.g. rate constants, Michaelis-Menten constants and kinetic orders). Furthermore, the population size is an important parameter as it governs the observed time courses of acids and solvents.

As a mechanistic approach, dynamic models rely heavily on estimation of their parameters from experimental data. This is a non-trivial numerical problem (e.g. Ashyraliyev et al. 2009; Chou and Voit 2009), as parameter identification is strongly affected by quality and quantity of experimental data and experimental design (Audoly et al. 2001; Banga et al. 2002; Emery and Nenarokomov 1998). Due to these restrictions, reported dynamical models of ABE fermentation consider only the central metabolic reactions of the network, i.e. those that describe the conversion of sugars, often glucose, to acids and solvents. Additionally, consecutive steps in the conversion of key metabolites are often combined into single steps to further simplify the system.

In vitro biochemical studies have elucidated the mechanisms of many enzymatic reactions in the ABE network (see Gheshlaghi et al. 2009 and references therein). Based on this information, Michaelis-Menten-like constants had been determined for a majority of reactions. However, the lack of data for catalytic constants (or limiting rates if multiplied with the total enzyme concentration) creates a serious problem for dynamical models, because the dynamic behaviour is encoded in these parameters and not the Michaelis-Menten constants (Millat et al. 2013b). The importance of limiting rates might be illustrated by the following: solventogenic clostridia are able to regulate limiting rates by changing enzyme concentrations, whereas Michaelis-Menten constants are solely determined by physical properties. Hence, experimental determination of limiting rates, i.e. catalytic constants and enzyme concentrations, is crucial for an improved understanding of dynamic changes observed in ABE fermentation.

It should be noted that, similar to structural models, dynamical models have to obey physicochemical and biological constraints, e.g. the computed metabolite concentrations have to be non-negative and physiologically reliable, catalytic constants have to be non-negative and smaller than the diffusion limit, etc. Furthermore, the network structure is also defined by a stoichiometric matrix.

The irreversible decarboxylation of acetoacetate by acetoacetate decarboxylase (inconsistently abbreviated as Adc or Aadc), which results in formation of acetone and carbon dioxide formation, illustrates the requirement for improved consideration of both kinetic and genetic regulation. Its catalytic ‘constant’, *k*_cat_, exhibits a remarkably strong pH dependence in the physiological range of clostridial ABE fermentation (Andersch et al. 1983; Ho et al. 2009). Here, conclusions about the metabolic activity of the reaction based solely on transcriptomic or (preferably) proteomic data might be misleading.

In the absence of such detailed information, the measurement of the ratio of catalytic constant and Michaelis-Menten constant would provide valuable information, because enzyme kinetic reactions can also be represented as apparent bimolecular reactions that are only determined by this ratio (Millat et al. 2007).

### Dynamic models describing batch cultures

In the late 1980s, Volesky and co-workers published a series of dynamic models for ABE batch fermentation using a modified *C. acetobutylicum* ATCC 824 (Votruba et al. 1986; Yerushalmi et al. 1986a; Srivastava and Volesky 1990). Their initial model (Votruba et al. 1986) consisted of a set of differential equations describing product formation, acid re-assimilation and sugar consumption, without considering intracellular metabolites. The authors termed this model process-oriented, but referred to it as ‘process kinetics model’ in a later publication (Yerushalmi et al. 1986a). A basic assumption of the model was that acid formation is inhibited by butanol, which resulted in increased solvent formation. It also incorporated re-assimilation of acetate and butyrate and their immediate conversion into ethanol and butanol, respectively.

Fermentation metabolism was directly coupled to growth rate which in turn depended on the available extracellular sugar and was inhibited by butanol. Additionally, the authors assumed that cellular decay occurs because of butanol poisoning. To represent biomass growth, the model employed the ‘concept of physiological state’ (Harder and Roels 1982) that correlates specific growth rate and RNA content.

Based on the process kinetics model, Volesky and co-workers subsequently developed a ‘physiological state model’ (Yerushalmi et al. 1986a) that distinguished between intra- and extracellular concentrations of acids and solvents. Here, intracellular product formation was described by the original process kinetics model (Votruba et al. 1986). The intracellular product concentrations were coupled to their extracellular counterparts via diffusion along concentration gradients. Additionally, acid transport was facilitated along an electrical potential gradient generated by the dissociation of acids in the extracellular medium (Yerushalmi et al. 1986b). Furthermore, a carrier model (Weiss 1996) was used to describe the transport of sugar into the cells, in which transporter activity was assumed to be inhibited by butanol.

Major criticism (Ataai and Shuler 1988; Bajpai and Iannotti 1988) of both models arose from the fact that they were insensitive to variations in the culture pH. Because the culture pH crosses the p*K*_a_ values for acetate (p*K*_a_ = 4.76) and butyrate (p*K*_a_ = 4.82), it is generally assumed that their conversion into solvents is a protective countermeasure against undissociated acids diffusing back into the cells. In an updated physiological state model (Srivastava and Volesky 1990), the pH-dependent dissociation of acids was therefore considered using the Henderson-Hasselbach equation (Atkins and de Paula 2002).

To identify important process parameters, the authors conducted a parametric sensitivity analysis showing that biomass growth and butanol and butyrate accumulation exhibited the highest sensitivity and, thus, represented the most important process parameters. The next highly ranked parameters determined the efficiency of transmembrane transport processes, i.e. sugar transport into the cell and product transport out of the cell, leading to the hypothesis that alterations in transport coefficients could improve ABE fermentation (see Yerushalmi et al. 1986a for further details and experimental hypothesis testing).

Despite their interesting results, the above models share a major drawback linked to their origin as process-oriented models: the structure of the ABE network and features of network components were completely neglected. Furthermore, ^14^C-labelling experiments have shown that a significant portion of re-assimilated acetate is also converted to butanol (Wood et al. 1945), which disagrees with the acetate re-assimilation mechanism assumed in the models which leads exclusively to ethanol. Moreover, pH-dependent regulation of enzymes, including their kinetic properties, gene expression and cellular abundance, were not considered. Finally, the inhibitory role of butanol is challenged by experiments indicating that acetate and butyrate have a stronger inhibitory impact on cell growth than butanol (Yang and Tsao 1994; Ballongue et al. 1987).

A dynamic model of ABE fermentation in *C. saccharoperbutylacetonicum* N1-4 ATCC 13564 was introduced by Shinto et al. in 2007 (Shinto et al. 2007). For the sake of simplification, formation of products from their corresponding branch metabolites was represented by single conversions. Furthermore, the authors assumed reversible reactions for both acetate and butyrate formation in addition to CoA transferase-mediated acid re-assimilation, even though there is experimental evidence contradicting this assumption for acetate formation (Andersch et al. 1983; Hartmanis et al. 1984). In the model, all metabolic reactions were coupled to a biomass function which in turn was determined by a cellular growth function and a cellular death function. The growth of the culture was assumed to be proportional to the formation of acetyl-CoA with the result that ABE fermentation and biomass formation competed for this key intermediate. Increasing acid concentrations initiated their re-assimilation and butanol formation. In addition, the formation of butanol was assumed as self-inhibitory.

The model was fitted to data obtained for batch cultures starting with an initial acetate concentration of 40 mM—a value usually observed at the final stage of standard batch culture experiments—which renders a comparison to other experimental and theoretical results difficult. Furthermore, several experimental findings were not taken into account, e.g. constant kinetic parameters and an acetone production independent from the metabolic state do not agree with experimental evidence. Furthermore, the model also predicted lactate formation in approximate same amounts as ethanol under normal fermentation conditions, which is in contrast to experimental findings (Jones and Woods 1986).

In the second part of the paper, a single-parameter sensitivity analysis with respect to butanol formation was conducted. However, the results of this analysis are of only limited informative value, as several kinetic parameters in the ABE network depend on each other. For instance, reactions that are carried out by the same enzyme (e.g. ethanol and butanol formation by AdhE2 or acetate and butyrate activation by CtfAB) could be affected simultaneously.

Similar modelling approaches were applied in two subsequent publications. In the first (Shinto et al. 2008), Shinto and co-authors compared the effect of glucose and xylose (via the pentose phosphate pathway) utilization rates when used as sole carbon and energy source by *C. saccharoperbutylacetonicum* N1-4. Interestingly, the study concluded that slower substrate utilization gives rise to higher butanol yields and, thus, slower xylose utilization resulted in higher product yields. However, any regulatory effects of butyrate and butanol on butyrate re-assimilation and butanol formation were not considered in this model, in contrast to the preceding studies discussed above.

Li et al. (2011a) used a similar but extended model, which considered two sequential reactions for the formation of acetate and butyrate, to investigate the role of butyryl phosphate in the initiation of solvent formation in *C. acetobutylicum*. Interestingly, the computed time course of butyryl phosphate reproduced the experimentally observed two peaks in intracellular butyrate phosphate levels, which correlate to the initiation of solvent formation and butyrate re-utilization, respectively. However, the qualitative behaviour of predicted butyryl phosphate, butyrate and butanol concentrations deviated from the reported data (Zhao et al. 2005). This suggests that the actual mechanisms for acetyl/butyryl phosphate formation differ from those assumed in the model. Indeed, experimental findings for *buk* and *pta* mutants had already suggested that two different mechanisms were responsible for the observed double-peak structure (Zhao et al. 2005).

In a more recent study, Raganati et al. (2015) investigated the effects of different sugars in ABE batch fermentations of *C. acetobutylicum* by applying an updated version of the model introduced in Shinto et al. (2008). In this updated model, a formally unbounded butanol inhibition of cell growth was replaced with a finite ‘critical’ butanol concentration which also constrained butanol-inhibited glucose uptake and self-inhibitory butanol formation. Similarly, critical concentrations were introduced for acetate, butyrate, acetone and ethanol, leading to an improved biomass equation that considered inhibitory effects of all liquid fermentation products. The resulting estimated parameter values principally agreed with previous results (Yang and Tsao 1994) showing that acetate, butyrate and butanol significantly inhibit growth, although their ranking remained uncertain.

As might be expected, sugar uptake activities were found to be strongly dependent on the given substrate and to coincide with experimentally confirmed preferences. Furthermore, cultures fed with glucose possessed the highest metabolic activity and the lowest tendency to sporulate. However, rates of other investigated hexoses differed from glucose only within a 20% range. In accordance with early studies of clostridial ABE fermentation (Robinson 1922), the authors found that fermentation of xylose and arabinose is distinct from that of other sugars. Furthermore, they speculated that the slower utilization observed for disaccharides, sucrose and lactose resulted from different membrane transport mechanisms and hydrolysis reactions. Although the updated batch culture model (Raganati et al. 2015) provided improved insights into ABE fermentation of different sugars, a sophisticated understanding of the reported findings based on their biochemical and thermodynamic properties remains elusive.

Taken together, existing dynamic models of ABE batch fermentation focus almost exclusively on the metabolic level. As a result, detailed metabolic models had been developed which consider the involved biochemical reactions as functions of metabolite concentrations, including positive and negative product regulation. Butanol in particular is assumed to inhibit cellular activity. However, given that all models predict butanol formation considerably earlier than is experimentally observed, this assumption must be questioned. Additionally, dynamic batch models commonly link the metabolic shift to sugar depletion, whereas it is experimentally well established that the majority of substrate (glucose) is converted following the shift to solvent formation and that sugar-limited batch cultures do not exhibit the classical ABE fermentation profile (Monot and Engasser 1983; Long et al. 1984).

Furthermore, changes in transcriptome, proteome and endo metabolome, which have obvious effects on cellular metabolic activities and parameters, as well as some alterations in the environome (in particular the pH level) are barely considered. While this information was not available when the first series of batch models was developed during the mid-1980s, it has not been taken advantage of even in the most recent batch culture models. As a consequence, despite yielding some interesting results, currently available batch models ultimately fail to provide a mechanistic, biological explanation for the metabolic shift from acid to solvent formation.

### Dynamic models describing continuous cultures

Continuous culture allows bacteria to be grown in a constant, well-defined and highly reproducible environment. Important factors such as the concentration of nutrients and fermentation products, pH value, growth rate and population density, which inevitably change during batch culture growth, are maintained at a steady state and may even be varied independently (Herbert et al. 1956) In comparison to traditional ABE batch culture, continuous culture thus allows a better separation of environmental and cellular changes and, therefore, the complex cascade of events finally resulting in the metabolic shift from acid to solvent formation. Despite these advantages, dynamic models describing continuous cultures have only been developed relatively recently as a result of a systems approach initiated by the transnational European COSMIC consortium to investigate ABE fermentation and sporulation in *C. acetobutylicum*.

All experiments and targeted mutations used a derivative of *C. acetobutylicum* ATCC 824 (the ‘COSMIC strain’) originating from the Rostock strain collection. ABE fermentation was studied using phosphate-limited continuous culture which offered superior reproducibility over traditional batch cultures (Hoskisson and Hobbs 2005). In particular, the consortium focussed on investigating the cellular changes caused by altering the culture pH from pH 5.7 (acidogenic state) to pH 4.5 (solventogenic state), termed ‘forward-shift’, and from pH 4.5 to pH 5.7, termed ‘reverse shift’ (for further details about the experimental setup and sampling methods, see Fischer et al. 2006; Fiedler et al. 2008). The obtained experimental data was exploited in several continuous culture models of the pH-induced metabolic shift which will be discussed in the following paragraphs.

Common to all models is their focus on intra- and extracellular changes and their effects on the metabolic activity of the culture. A simplified metabolic model of ABE fermentation was applied that included all key branch metabolites (acetyl-CoA, acetoacetyl-CoA and butyryl-CoA) and the major non-gaseous fermentation products. Assuming a quasi-steady state, intermediary steps between those metabolites were merged into a single conversion step. In contrast to the dynamic batch culture models discussed above, additional biological data based on proteome analyses were taken into consideration.

The first model published by Haus et al. (2011) studied the observed pH-induced changes in gene expression with a focus on the two steady states, i.e. at pH 5.7 and pH 4.5. Because previous experimental studies (Janssen et al. 2010; Grimmler et al. 2011) observed no significant changes in the cellular abundance of acid-forming proteins at acidogenic (pH 5.7) and solventogenic (pH 4.5) steady states, only the synthesis of solvent-forming proteins was assumed to be coupled to a pH-dependent switching function. A comparison of numerical simulations of forward and reverse shift experiments confirmed that the pH-induced metabolic switch involves an adaption of the proteomic composition, notably solvent-forming proteins, with clostridial cells promoting either acid or solvent formation.

Furthermore, the authors used their model to investigate the effects of targeted single gene induction, including upregulation and downregulation, on solvent formation at steady state. This numerical analysis found that the regulation of a single solvent-forming gene is insufficient to significantly increase butanol formation. For instance, the model predicted that butanol production cannot be markedly improved by simply overexpressing the *bdhA*/*B* (butanol dehydrogenase) genes. Interestingly, considerable upregulation of *adhE*1, believed to be involved in the formation of both ethanol and butanol, was predicted to have a detrimental effect on formation of the latter, because formation of ethanol was preferred instead. However, a slight overexpression was predicted to have a positive effect on butanol formation. This result indicated that traditional sensitivity analysis focussing only on small changes might be misleading. Moreover, it suggested that successful metabolic engineering of the ABE fermentation network might rely on tightly controlled overexpression and underexpression of the genes involved.

However, while this first model could explain the observed differences between acidogenic and solventogenic cells, it failed to reproduce the experimentally observed transition dynamics between the two metabolic steady states.

Thorn et al. (2013) therefore extended the model by also considering pH-dependent sporulation, the assumption being that acid-forming cells sporulate in response to the sudden drop in culture pH, resulting in metabolically inactive cells. This improved the fit to experimental data, although an optimal fit was only achieved for unrealistically large proportions of sporulating cells. Another focus of this investigation was on parameter estimation for ABE fermentation and how it could be improved by additional experimental data. This analysis revealed the need for quantitative experimental data for internal metabolites, in particular branch metabolites and reactions mediated by CtfAB CoA transferase.

Motivated by the first model’s (Haus et al. 2011) incapacity to accurately reproduce the transition between acid- and solvent-forming steady states, a systematic re-analysis of the dynamic shift experiments was conducted by Millat et al. (2013a). As a result, the authors developed a workflow for data analysis and data processing of pH shift experiments in continuous culture. A key point of this workflow was the analysis and representation of the measured optical density using a phenomenological fitting function. A significant but transient drop in OD during the metabolic transition has been consistently observed in forward shift experiments (Janssen et al. 2010; Grupe and Gottschalk 1992; Dürre et al. 1995). In Millat et al. (2013a), the authors interpreted this as indicative of a shift from an acidogenic to a solventogenic population which significantly differ in their transcriptomic, proteomic and, thus, metabolomic profiles.

As a consequence, the new shift model developed by Millat et al. (2013a) considered two subpopulations with distinct metabolic activity and assumed that acidogenic cells stop growing when the pH falls below 5.2 to 5.1 (Millat et al. 2013b) while, simultaneously, the solvent-forming cells increase in numbers and eventually establish a solventogenic culture. This pH-dependent growth is coupled to an updated metabolic model which takes into account the experimentally observed differential antagonistic expression of the bifunctional alcohol/aldehyde dehydrogenases AdhE1 and AdhE2 (Janssen et al. 2010; Grimmler et al. 2011). Furthermore, kinetic regulation was considered for those enzymes that are known to have a significant pH-dependent specific activity, notably acetate kinase, butyrate kinase and acetoacetate decarboxylase (Andersch et al. 1983; Ho et al. 2009).

Due to the consideration of multiple levels of biological organization, this model much better explained the experimental data obtained for the forward-shift experiments. In particular, it raised the question of whether the previously assumed homologous nature of ABE fermentation should be reconsidered.

Model-derived formation rates for acetate and butyrate in forward-shift experiments suggested that there is a significant re-assimilation activity regarding butyrate, but not for acetate. This agrees with results obtained for batch cultures (Mermelstein et al. 1993) but is in contrast to biochemical studies on CtfAB CoA transferase (Hartmanis et al. 1984) which showed a higher activity of this enzyme with acetate. Moreover, the model required that few solventogenic cells exhibit an extremely high assimilation activity, because considerable butyrate uptake occurred at a stage when the postulated solventogenic subpopulation was still small.

These issues were addressed in a follow-up study (Millat et al. 2014), which examined the behaviour of a *ctfA* mutant using the same experimental setup and model as previously for the wild type. As expected, this mutant was incapable of forming acetone but it still re-assimilated butyrate. Using the model established in Millat et al. (2013b), but assuming an inactive CoA-transferase, it was shown that pH-dependent growth and continuous wash-out alone were insufficient to reproduce the time course observed for butyrate whereas predictions agreed well with the observed acetate concentrations.

Butyrate assimilation in the absence of CoA transferase could not be explained without the addition of a pathway that converts butyrate to butyryl-CoA into the model. The thus updated model (Millat et al. 2014) predicted this mechanism to be pH-dependent, shifting the equilibrium towards butyryl-CoA formation for low pH levels. Additionally, the calculated relative change in butyrate formation rate agreed well with the relative changes in specific activity of butyrate kinase reported in Andersch et al. (1983). Thus, it was concluded that a CoA transferase-independent mechanism is indeed at work and responsible for the assimilation of butyrate, but not acetate, during the metabolic shift.

Unfortunately, these findings were later misinterpreted in recent publications (Croux et al. 2016; Yu et al. 2015). Contrary to statements made in Croux et al. (2016) and Yu et al. (2015), the work undertaken in Millat et al. (2014) provides no proof of the roles of butyrate kinase (Buk) and phosphotransbutyrylase (Ptb) in butyrate re-assimilation, as it allowed for other mechanisms. Indeed, the authors of Millat et al. (2014) proposed the combined action of aldehyde/ferredoxin oxidoreductase and butanol dehydrogenase as a possible alternative.

Very recently, Thorn and King (2016) applied the workflow and model introduced in Millat et al. (2013a) on data reported for a reverse shift experiment (Haus et al. 2011), in which the external pH of a continuous culture was shifted from a low level (pH 4.5) to a higher level (pH 5.7). Published accounts of this type of shift experiment are rare, and thus, substantially less information is available. Since Haus et al. (2011) provided no optical density data, Thorn and King assumed that the acid- and solvent-forming subpopulations exhibit a behaviour that mirrors the forward shift. After re-estimating the model parameters for a reverse shift, the authors found that a constant amount of CtfA/B CoA transferase provided the best fit to the available data, although this contradicted the notion of CtfAB as a solventogenic enzyme.

In summary, modelling ABE fermentation for continuous cultures has corroborated the view that the metabolic shift in solventogenic clostridia is orchestrated by a complex network of interactions involving all levels of biological organization. While the existing models consider regulation at the kinetic, cellular and population level in a phenomenological manner, they do not establish the causal relations that exist between them. For instance, the observed drop in culture pH is described by a mathematical function independent of the cellular metabolic activity which actually causes it. Furthermore, while these models assume co-existing acidogenic and solventogenic populations, they provide no insight as to how, when and why these emerge.

## Conclusions and outlook

Modelling ABE fermentation has a 30-year history, starting in 1984 with the very first application of flux balance analysis to a structural model. Since then, mathematical modelling has increasingly contributed to our understanding of clostridial ABE fermentation. Research activities in this area have primarily focussed on two aspects, first to understand the regulation and cellular alterations resulting in the reversible shift from acidogenesis to solventogenesis and, second, to improve production of butanol and acetone. The latter includes bioreactor and process design, which were not considered in this review, but also genetic modification and metabolic engineering.

In accordance with experimental data obtained for batch and continuous cultures, structural and dynamic models have confirmed that the transition from acidogenesis to solventogenesis requires a drastic shift in the activity of the different fermentation pathways involved. This shift is accompanied by considerable changes in cellular composition, suggesting that this adaption cannot solely be explained by kinetic regulations, e.g. the inhibitory effects of metabolic products. Recent experiments have shown that transcriptome, proteome and metabolome are linked in a complex, sometimes non-intuitive, manner that is only incompletely understood. These interactions will have to be established in upcoming models, and this very likely requires better knowledge of the kinetic properties of the enzymes involved.

Furthermore, the experimentally observed dependence of product spectrum and temporal behaviour of clostridial ABE fermentation upon substrate and intracellular pools of ATP and NAD(P)H offers challenges for future models. Stoichiometric considerations already indicate that balancing substrate and products results in different product yield depending on how many atoms of carbon, hydrogen and oxygen (often described as state of reduction) a substrate is able to provide (Papoutsakis 1984; Johnson et al. 1931). An understanding of the different behaviour observed for sugars with identical chemical formulae (e.g. glucose, mannose, fructose) is likely to require the consideration and comparison of kinetic and thermodynamic properties at all levels of cellular organization.

Interestingly, some of the modelling attempts outlined above challenge the classical view of ABE fermentation. For instance, recent theoretical findings suggest that the metabolic shift might be considered a heterogeneous phenomenon, where acidogenic and solventogenic (and potentially other) phenotypes represent different stable states of the metabolic network (Millat et al. 2013a; Senger and Papoutsakis 2008b; Kumar et al. 2014). If so, the observed metabolite spectrum may be calculated as the sum over the metabolic activity of those phenotypes.

$$ \left[\begin{array}{c}\hfill \frac{dx_1}{dt}\hfill \\ {}\hfill \vdots \hfill \\ {}\hfill \frac{dx_m}{dt}\hfill \end{array}\right]=\sum_p{\mathcal{N}}_p\cdot {\left[\begin{array}{ccc}\hfill {s}_{11}\hfill & \hfill \cdots \hfill & \hfill {s}_{1 n}\hfill \\ {}\hfill \vdots \hfill & \hfill \ddots \hfill & \hfill \vdots \hfill \\ {}\hfill {s}_{m1}\hfill & \hfill \cdots \hfill & \hfill {s}_{m n}\hfill \end{array}\right]}_p\cdot {\left[\begin{array}{c}\hfill {\nu}_1\hfill \\ {}\hfill \vdots \hfill \\ {}\hfill {\nu}_n\hfill \end{array}\right]}_p- D\cdot \left[\begin{array}{c}\hfill {x}_1\hfill \\ {}\hfill \vdots \hfill \\ {}\hfill {x}_m\hfill \end{array}\right]. $$

Here, the subscript *p* denotes different phenotypes, which might represent one of the following two principal phenotypic classes. First are ‘multistable’ phenotypes, where cells populate different optima of an identical network. This behaviour is well known in the stochastic theory of multistable systems (Ebeling and Sokolov 2005; van Kampen 2007; Schlögl 1972). Most of the existing structural models, including genome-scale models, have so far neglected this feature, but would in principle be capable of identifying multiple optima and their flux distributions (Wintermute et al. 2013). Second are ‘selective’ phenotypes, where cellular regulation activates (or deactivates) only parts of the network. Thus, these phenotypes differ in their network structure and stoichiometric matrices. Mathematical models of bacterial metabolisms often consider such changes in network structure by manually employing switching functions or by assuming distinct phenotypes. Note that selective phenotypes might also exhibit multiple stable solutions.

Indeed, over the years, experimental evidence has repeatedly emerged to suggest that ABE fermentation may involve heterogeneous populations. For instance, for continuous cultures, several groups observed oscillations in the levels of acids and solvents (Clarke et al. 1988; Roos et al. 1985) or the redox potential (Kim and Kim 1988; Grupe and Gottschalk 1992). Furthermore, spectroscopic single-cell analysis using Raman microscopy implied that cells growing in batch culture also exhibit heterogenic features (Schuster et al. 2000b).

Thus, the identification and characterization of phenotypically different subpopulations is an important task for future studies, both experimental and theoretical. The latter might reveal the mechanisms behind the emergence of such phenotypes and their relative abundance in mixed populations as a consequence of their differing abilities to conserve energy under the prevailing conditions. Shifting the ratio in heterogeneous population towards desirable (e.g. butanol-producing) phenotypes might offer an interesting alternative to the traditional engineering of metabolic networks.

Considering ABE fermentation as a multistable system may also reveal an interesting feature not commonly associated with metabolic processes. In a multistable system, the system’s history can affect or even determine its future developmental options and capabilities. Thus, a sequence of metabolic states might be interpreted as developmental process (e.g., Veening et al. 2008; Ackermann 2015).

The existence of a solventogenic phenotype (or, more likely, phenotypes (Schuster et al. 2000b; González-Peñas et al. 2015; Tracy et al. 2008)), which can grow and divide, would challenge the long-standing notion of a non-growing solventogenic state. If confirmed, solventogenic metabolism would be subject to constraints different to those applied in some of the existing models. Models might need to distinguish between acidogenesis, alcohologenesis (Girbal et al. 1995; Meyer et al. 1986; Vasconcelos et al. 1994), solventogenesis and potentially other states.

Despite the substantial contribution of mathematical models to our understanding of clostridial acetone-butanol-ethanol formation, they are yet unable to pinpoint definitive causes and mechanisms. It is evident that an integrated multi-level approach, including transcriptomics, proteomics and metabolomics, as well as information regarding the environoment, kinetic regulation and population heterogeneity, will be required to shed light on the process. A focus on the individual ‘objectives’ of cells and populations might also prove beneficial. Doing so will bring about new experimental and theoretical approaches, hopefully leading to a sustainable biotechnological process in the future.
